# Behavioral Competence as a Positive Youth Development Construct: A Conceptual Review

**DOI:** 10.1100/2012/568272

**Published:** 2012-05-02

**Authors:** Hing Keung Ma

**Affiliations:** Department of Education Studies, Hong Kong Baptist University, Hong Kong

## Abstract

Behavioral competence is delineated in terms of four parameters: (a) Moral and Social Knowledge, (b) Social Skills, (c) Positive Characters and Positive Attributes, and (d) Behavioral Decision Process and Action Taking. Since Ma's other papers in this special issue have already discussed the moral and social knowledge as well as the social skills associated in detail, this paper focuses on the last two parameters. It is hypothesized that the following twelve positive characters are highly related to behavioral competence: humanity, intelligence, courage, conscience, autonomy, respect, responsibility, naturalness, loyalty, humility, assertiveness, and perseverance. Large-scale empirical future studies should be conducted to substantiate the predictive validity of the complete set of these positive characters. The whole judgment and behavioral decision process is constructed based on the information processing approach. The direction of future studies should focus more on the complex input, central control, and output subprocesses and the interactions among these sub-processes. The understanding of the formation of behavior is crucial to whole-person education and positive youth development.

## 1. Background

 The goal of education is not only to teach our children the knowledge and skills to behave gracefully and properly but also to act accordingly. In other words, education should not stop at the judgment level but should also go straight to the real behavior domain. The development of behavioral competence is therefore one of the most important attributes that we want to teach students. In this paper, we will explore a set of positive characters or attributes that are highly related to behavioral competence. In addition, the behavior decision process is investigated based on the information processing approach. We are interested in studying the factors affecting one's behavioral decisions, especially in dilemma situations.

## 2. Definition of Behavioral Competence

 Behavioral competence can be defined in terms of four parameters: (a) Moral and Social Knowledge: children should learn the social norms, common manners and rituals, social laws, rules, and regulations in an organization or group (e.g., home, school, government, company, or interest group), and the sociomoral values in one's society. This parameter is explained in detail in Ma's [1] paper on moral competence. (b) Social Skills: this refers to the ability to use verbal and nonverbal strategies to perform socially, morally acceptable and normative behavior in daily interaction with others [2, 3]. “Verbal strategies mainly include oral expression and writing, whereas nonverbal strategies include body language such as facial expression, tone of voice, gesture, and eye contact” [2, page 177]. The social skill parameter is delineated in Ma [2] as well as his paper on social competence in this special issue [3]. (c) Positive Characters and Positive Attributes: the development of behavioral competence is highly related to positive characters (e.g., respect, responsibility, humanity, and loyalty) and prosocial orientation or motivation (e.g., courage, perseverance, and conscience). Positive characters promote moral behavior, and positive motivation triggers and sustains good actions. (d) Behavioral Decision Process and Action Taking: the whole process of making a behavioral decision and finally realizing the decision by carrying it out could be a highly complicated procedure. The present paper focuses on the last two parameters, and an information processing approach is explored here.

## 3. Positive Attributes Related to Behavioral Competence

 There are many positive attributes related to behavioral competence. Ma [4] proposed that there are ten primary moral characters that we need to help children to develop. These moral characters are humanity, intelligence, courage, conscience, autonomy, respect, responsibility, naturalness, loyalty, and humility. All these characters can be regarded as positive attributes that are closely related to behavioral competence. Two other attributes, assertiveness and perseverance, are also highly related to behavioral competence and will therefore be included here. The summary of the first ten characters is based on Ma's Model of Moral Character Education. A more detailed description is given in Ma [4]. The humanity (Jen), intelligence (moral cognition and moral judgment), conscience, and naturalness characters have been discussed in detail in Ma's [1, 5] theory of moral development for the Chinese people. In addition, the autonomy, respect, and responsibility characters were also delineated in Ma's [2, 3] study on social competence.

**Humanity**

*Jen* or humanity “has something of the love which parents have naturally for their children. It has something of the compassion which a man of sensitivity feels when seeing an innocent animal slaughtered” [6, page 27]. A great Confucian philosopher, Mencius, also said, Jen “is a feeling common to all mankind that they cannot bear to see others suffer" [6, page 132]. Humanity includes empathy, caring, and forgiveness. This feeling of profound love is the basis of genuine altruism and caring for others in all the social actions. The goal of education is to foster the development of Jen or humanity in our next generation so that they would love others as they love themselves.

**Intelligence**

It refers to the ability to adapt to difficult and complicated situations in a changing environment and making prosocial actions promptly and effectively in response. Many psychologists (e.g., J. Piaget and R. Sternberg) regard intelligence as the most important adaptive ability in one's development [7, page 203], [8, 9]. Students should therefore be taught to be critical, creative, rational, fair, and forgiving in their judgment and behavioral decision process in order to more effectively adapt to their surrounding environment. While intelligence appears to be a necessary condition for making a prosocial or moral decision, by itself it is simply not sufficient. Other factors such as moral emotion, prosocial motivation, and situational variables also affect the actual behavioral outcomes [5, 10, 11].

**Courage**

It is the emotional disposition and motivation that would push one to act, to move forward, and to do good and right things despite a dilemma, great difficulty, and serious threat. The vigorous fortitude and enduring sacrifice associated with the demanding course to uphold long-lasting courage can be quite overwhelming and unbearable to many common people. Courage is a core moral quality regarded by many moral philosophers [12]. The courage to act prosocially and refrain from temptation and corruption is a character that we should help children to develop.

**Conscience**

There are two major aspects of conscience: the cognitive and affective aspects [13, pages 57-58]. Cognitively, conscience knows what is right and what is wrong, and what one should do and what one should not do. The affective aspect refers to the feeling of shame and guilt when one has done something wrong or when one is not able to do something good or right. The conscience is quite related to the concept of morality defined by psychoanalytic psychologists, that is, one's moral action is motivated by a negative wish to avoid the painful feeling of shame and guilt [14]. The development of a moral conscience is another big concern of educators. A student with a high level of intelligence but a low level of conscience may not have positive and pleasant social interactions with others.

**Autonomy**

It should be noted that the concept of autonomy is a key value in Western cultures. Many psychologists and educators [5, 11, 15–17] regard genuine personal autonomy as an important characteristic of the highest stage of moral judgment. Based on their free will, autonomy, and freedom, people at this stage act according to their self-chosen universal ethical principles. In other words, people at such a high stage of moral development are able to transcend or resolve the moral disputes between the majority and an individual in a just and least conflicting manner. The universal ethical principles are the principles that are based on good will and are applicable to any person in any situation without exception.

**Respect**

According to Lickona [13], respect “takes three major forms: respect of oneself, respect for other people, and respect for all forms of life and the environment that sustains them” (page 43). Respect of oneself or self-respect refers to one's disposition to cherish one's worth and one's confidence in facing challenges. Respecting others stands for being polite, tolerant, graceful, dignified, sincere, honest, fair, humble, and caring of other peoples' feelings. The respect for all forms of life includes the respect for animal rights and the respect for the rights of all living things. It also includes a heartfelt respect and love for Nature and hence will cause one to try one's best to protect Nature.

**Responsibility**

The value of responsibility is derived from the value of respect and it includes four different perspectives [13]: (a) personal responsibility: it refers to one's reliability and trustworthiness; (b) familial responsibility: it prescribes people to be responsible and accountable for the welfare and protection of members of their family (e.g., one's parents, children, siblings, and spouse); (c) social and civil responsibility: a citizen has “the obligation to perform certain duties, including the responsibility to obey the law, to pay one's taxes, to respect the rights of other people, to fight for one's country, and generally to fulfil one's social obligations” [18, page 2]; (d) global responsibility: As a world citizen, one has responsibility to take care of the welfare of the people in their own countries and also people in other countries [3].

**Naturalness**

Chinese Tao philosophers place emphasis on naturalness (e.g., a childlike heart and adult intelligence) in the development of characters [19]. In the description of the characteristics of self-actualizers, Maslow [20] also argued that self-actualizing people always behave with a high degree of spontaneity, simplicity, and naturalness, which are common in young children's behavior. In short, the virtue of naturalness means purity, simplicity, softness, spontaneity, sincerity, and genuineness [21]. Therefore, the teaching of a pure, simple, sincere, and natural lifestyle should be beneficial to our next generation who are supposed to live in a sophisticated, complicated, and technological world when they grow up.

**Loyalty**

The virtue of loyalty is regarded as one of the core moral qualities in Confucianism [19]. To be loyal to a group means that one identifies with the value and interests of the group and would stand up to defend the interests of the group when the group is facing crisis or threats at the expense of one's personal interest. The identification with and loyalty to one's country favours the development of national patriotism [2]. The characteristics of loyalty are meant to be related to those of integrity and honesty.

**Humility**

We should be humble and polite towards others. Confucius once said “Behave with great respect and prudence when away from home as though you were receiving a distinguished guest. Preside over the common people with gravity and seriousness as though you were officiating at a grand sacrifice. Do not do to others what you would not want others to do to you” [22, page 193]. The teaching of humility in our children should hopefully reduce their aggression towards and any conflicts with their peers and adults. It is beyond doubt that a society of the humble and polite will be a society of peace, dignity, and happiness.

**Assertiveness**

Goleman [23] in his elaboration of emotional literacy argued that assertiveness “emphasizes expressing feelings forthrightly, but in a way that will not spiral into aggression” (page 266). Bonham-Carter [24] described assertive people as someone who “express their views clearly and articulately without being aggressive; stands up for their own and other people's rights in a reasonable and clear way; and allows other people a reasonable opportunity to express their opinions without allowing them to dominate a conversation.” In other words, children should be taught to defend their value and beliefs in a reasonably elegant and graceful manner. In particular, they should be able to debate with other people, especially aggressive and impolite people, on what they think is good and right. In a review of prosocial development, Eisenberg et al. [25, page 691] concluded that assertiveness is associated with prosocial behaviors in children. Assertive children are relatively high in sympathetic and prosocial behavior.

**Perseverance**

It refers to one's persistent effort to stick to one's aim or purpose and never give up in the face of difficulties and challenges. Two of the Chinese idioms are relevant here: “dripping water penetrates the stone” and “constant perseverance yields success” [26]. The concept of perseverance is well explained in the Analects of Confucius in several occasions. Two of them are presented here: (a) the philosopher Zeng said, “The officer may not be without breadth of mind and vigorous endurance. His burden is heavy and his course is long. Perfect virtue is the burden which he considers it is his to sustain—is it not heavy? Only with death does his course stop—is it not long?" [27, Tao Bo 7]. Scholars and officers must be strong and persistent. Their responsibility is heavy and the road ahead is long. They have the responsibility to practice and maintain perfect virtue until their death. Is the burden too heavy and is the course too long? (b) The Master Confucius said, “The superior man does not, even for the space of a single meal, act contrary to virtue. In moments of haste, he cleaves to it. In seasons of danger, he cleaves to it." [27, Li Ren 5]. The superior man should have the perseverance to act according to perfect virtue in any difficult, poor, deprived, and dangerous situations. In summation, Confucianism teaches us to act morally and to stick to perfect virtue with a perseverant personality. A similar concept of perseverance in personality psychology is “grit.” Duckworth et al. [28] defined grit as “perseverance and passion for long-term goals. Grit entails working strenuously toward challenges, maintaining effort and interests over years despite failure, adversity, and plateaus in progress” (pages 1087-1088). In the empirical study with two adult samples, they found that grit is positively related to success outcomes including educational attainments. Grit was also highly related to conscientiousness in the Big Five personality traits (the other four personality traits in the Big Five are: extraversion, agreeableness, neuroticism, and openness to experiences) [29]. “Conscientious individuals are characteristically thorough, careful, reliable, organized, industrious, and self-controlled” [28, page 1089]. In other words, grit or perseverance is related to carefulness, reliability, industry, and self-control.

There are two important features that we need to take note of in fostering the development of the above positive characters: (a) some of the above characters may not be of the same nature. For example, naturalness and humility may be at odds with assertiveness. How can one be simple, sincere, humble, and yet be assertive? The fact is that reality is very complicated. Our children may have to learn to adapt to a large variety of situations. In some situations, one has to be assertive at the beginning of the interaction and then turn to being humble later on. In the other situation, we may have to do the reverse, that is, humble first, assertive later. In addition, one may need to exhibit some of the characters in one situation and the other characters in the other situation. The complexity in the teaching of these characters is a big issue in curriculum development in moral education and whole-person education. (b) It is likely that some of the above characters are culturally specific. We propose these characters as a basis for teaching the Chinese children. Further researches have to be conducted to substantiate the cultural universality of these characters in the education of children in other cultures.

## 4. An Information Processing Approach to Behavioral Decision

 The behavioral decision process is studied by an information processing approach. We would focus on moral and prosocial behavior in the following discussion. According to Rest's [11] Model of Moral Judgment and Decision, the whole process consists of four components.

**(A) Interpreting the Stimulus and Identifying the Problem**

The incoming information passing through the Selective Filter of the Input System is interpreted by the organism. The interpretation may involve “imagining what courses of action are possible and tracing the consequences of action in terms of how each action would affect the welfare of each party involved” [11, page 5].

**(B) Formulating an Ideal Plan of Prosocial Action**

After the moral problem has been identified and structured, the next step is usually to formulate an ideal plan of action which satisfies one's ideals, values, or moral principles. In general, social norms affect one's formulation of a plan of moral action [11, pages 8–13]. Furthermore, the moral judgment level of an individual also affects the person to formulate a plan of action. People at a higher stage of moral judgment tend to formulate a plan of action based on democracy, respect of basic human rights, and principles of universal love and justice. On the other hand, people at a lower stage would act according to instrumental purpose, reciprocity, and expectations of their significant others [1, 17].

**(C) Evaluating Alternatives and Making Decisions**

Parallel to step (B), the actor will identify and evaluate all possible alternatives. It is quite common that the first possible action an individual thinks of is not a moral one or an ideal one. The evaluation of alternatives normally involves the assessment of the consequences of each alternative action and the pertinent uncertainties. Very often, people choose not to act according to their ideal plan of moral action. It may be due to one or more of the following factors: one's immediate feelings, the nature of the action, and one's social and moral values.

**(D) Output Process: Action Taking**

According to Broadbent [30, page 10], the output action is implemented by a set of effectors. It is obvious that the effect of the initial output action will be fed back into the organism in order to modify its further action. The reasons of why people very often cannot implement a moral plan successfully are quite complicated. Researches on ego strength, delay of gratification, and self-regulation (see, e.g., [31]) are relevant here. For example, whether a person is courageous or not, empathetic or not, intelligent or not will significantly affect the successful implementation of the moral plan.

Obviously, the model is not a linear one. It is quite true that the four components have a logical sequence: interpreting the stimulus, formulating an acceptable course of action, and executing, but interactions among the components are evident [11, pages 17-18].

 In order to equip students with the ability to implement one's moral choice in real-life situations, Raths, Harmin, and Simon have developed a model called “Values Clarification.” They proposed seven steps in valuing process: “choosing freely, choosing from alternatives, choosing after considering the consequences, prizing and cherishing, affirming, acting upon choices, and repeating” [32, pages 77-78]. The first three steps are related to component (C) “Evaluating Alternatives and Making Decisions” in the above model. The last four steps are related to component (D). The emphasis on cherishing and affirming of one's choice and repeating acting upon one's choice is essential in educating our children to act morally and prosocially. 

While the information processing approach provides a useful framework to study how a person processes incoming information, makes a behavioral decision, and eventually acts upon the decision, the details in each of the subprocesses require more sophisticated and scientific methods to work out. In addition, the detailed mechanism on how the environment influences the whole process is not yet fully understood. Finally, how the emotional state and the personality of a person affects the process is another interesting topic for further research. Despite the above limitations, the information processing approach becomes a popular approach to the study of learning [33].

## 5. Assessment of Behavioral Competence

 According to the definition of behavioral competency, the assessment of behavioral competence includes the assessment of the following: (a) Moral and Social Knowledge, (b) Social Skills, (c) Positive Characters and Positive Attributes, and (d) Behavioral Decision Process and Action Taking. Assessment of moral and social knowledge is discussed in several reviews or articles [1, 3, 25, 34, 35] and assessment of social skills can be found in Bashook [34], Eisenberg et al. [25], and Ma [2]. The proposed twelve positive characters require future empirical studies to substantiate their predictive validity of behavioral competence. As for the behavioral decision process, assessment of the variables and attributes of each component is difficult and usually less reliable and valid at the present stage [10, 11]. The discussion on the complete set of behavioral competences is complicated and is beyond the scope of this paper.

## 6. Antecedents of Behavioral Competence

The antecedents of behavioral competence are similar to those of moral and social competences [1, 3]. Parental and peer influences on behavioral competence are significant [25, 36, 37]. The following discussion will focus on teacher influences. Teachers exert significant impact on the development of behavioral competence in children [25, 38]. Epstein and his colleagues [38] in their review on problem behavior in elementary school children made the following five recommendations to help the students by reducing their problem behavior and increasing their appropriate behavior in schools. (a) “Identify the specifics of the problem behavior and the conditions that prompt and reinforce it” (page 14): a correct understanding of the main causes of the problem behavior is most important for choosing the right teaching strategies to help the students. (b) “Modify the classroom learning environment to decrease problem behavior” (page 22): teachers can reduce the problem behavior by removing the factors that trigger the undesirable behaviors (e.g., changing the classroom environment and teaching schedule and increasing learning activities that meet students' needs). (c) “Teach and reinforce new skills to increase appropriate behavior and preserve a positive classroom climate” (page 29): instead of focusing on problem behavior, teachers should teach new skills for appropriate behavior and withhold reinforcers for problem behavior. (d) “Draw on relationships with professional colleagues and students' families for continued guidance and support” (page 37): teachers should rely on guidance and support from professional colleagues such as senior teachers, school administrators, social workers, and counseling psychologists as well as students' parents in dealing with students' inappropriate behaviors in schools. (e) “Assess whether schoolwide behavior problems warrant adopting schoolwide strategies or programs” (page 44): in many cases, students' problem behavior can be reduced with the help of school personnel (e.g., school principal, discipline teacher, counseling teacher, and subject team). Consistent and transparent school policy and a positive school social environment are also essential in supporting the teachers to reduce inappropriate student behavior.

## 7. Relationship between Behavioral Competence and Adolescent Developmental Outcomes

 Behavioral competence tends to be related to cognitive competence, emotional competence, moral competence, and social competence.

 Cognitive competence is the basis of all behaviors. It includes the intelligence and the information processing ability in making behavioral decisions. Research indicated that intellectual ability as measured by the IQ scores predicts quite well the school achievements of children. On the other hand, the IQ scores do not appear to predict income and job success in later life [8, page 120]. Perhaps cognitive competence is a necessary condition for behavioral outcomes but not a sufficient condition. Emotions tend to “energize thinking and acting in ways that are often adaptive to the circumstances” [7, page 417]. Adolescents with emotional problems usually have difficulty in social interaction with others, for example, making friends with their peers. In other words, adolescents with high emotional competence tend to adapt to the social environment better than those with low emotional competence.

 Kohlberg and his colleague [39], [40, pages 498–581] argued that moral stage predicts moral behavior. Those at a higher stage of moral judgment tend to act more prosocially and morally than those at a lower stage. Moral development is associated negatively with antisocial and delinquent behavior [41, 42]. In a study of moral development and social behavior of Chinese adolescents in Hong Kong, Ma [43] defined moral development in terms of affective and altruistic orientation and moral judgment. He found that the moral orientation and moral judgment of the prosocial adolescents are higher than those of delinquent adolescents. In other words, prosocial behavior is positively associated with moral development and delinquent behavior is negatively associated with moral development. In general, adolescents with high-level moral competence tend to act more prosocially and less antisocially. Similarly, Eisenberg et al. [25] concluded in their review that prosocial behavior is positively associated with social competence. As mentioned before, assertiveness is associated with prosocial behavior in children [25] and perseverance predicts successful outcomes and educational attainment [28].

 It is hypothesized that each of the twelve positive characters is related to one or more of the competences: moral, social, emotional, cognitive, and behavioral. For example, intelligence is related to cognitive competence, humanity and courage are related to moral and emotional competences, and assertiveness and perseverance are related to social and emotional competences. In addition, all of these twelve characters are hypothesized to be positively related to behavioral competence. Albert Bandura argued that “a person's behavior is always the result of interactions among personal characteristics, behavioral patterns, and environmental factors” [33, page 270]. Based on this rationale, it is hypothesized that the twelve positive characters interact reciprocally with behavior. In other words, some of the characters may act as antecedents of a certain kind of behavior, which may also serve as antecedents of some other characters. Future studies should be conducted to work out the details of this reciprocal interactive mechanism between the twelve positive characters and behavior.

The information processing ability to identify incoming stimulus, to make an action choice after considering various alternatives, and to act upon the final choice is highly related to cognitive, social and moral competences [10, 11], which in turn are supposed to be related to behavioral competence as well. Future empirical researches are recommended to be carried out to substantiate these hypotheses systematically.

## 8. Practical Ways to Promote Behavioral Competence in Adolescents

 Apart from teacher's influence, school-based programs on positive youth development are also effective in facilitating students' behavioral competence development. One popular teaching package on positive youth development in Hong Kong is P.A.T.H.S. (Positive Adolescent Training through Holistic Social Programs). In an intervention study involving 3,006 experimental students and 3,727 control students, Shek and Ma [44] found that “participants in the experimental schools displayed better positive youth development than did participants in the control schools based on different indicators derived from the Chinese Positive Youth Development Scale, including positive self-identity, prosocial behavior, and general positive youth development attributes” (page 253). In a similar study on problematic behavior, Shek and Yu [45] also found that the participants from the experimental schools “displayed lower level of substance abuse and delinquent behavior than did the control students” (page 546). These findings clearly indicated that good school-based program is useful and effective in helping students develop behavioral competence.

 There are two rather radical attempts on implementing real action in schools: Lawrence Kohlberg's Just Community Approach and Fred Newmann's Social Action Model [32]. Democratic governance is the main focus of the Just Community Approach. Kohlberg has tried his approach in an alternative high school in 1974. About sixty students and six staff members from the regular school participated in this study. Students and teachers in the alternative school met once a week for a two-hour community meeting to decide on the policy and rules for running the alternative school. For example, what sort of punishment should be imposed on cutting classes, fighting, stealing, and using drugs? Members of the alternative school had the autonomy to adopt rules for running their own school. Once a rule was set up by the agreement of the majority of the students, all members had to comply with the rule and regulation. Violators of the rules were subject to disciplinary action by a discipline committee which comprised student representatives and teachers as members [32, pages 150–157]. In contrast, Fred Newmann's Social Action Model focuses on citizen action and “aims to teach students how to influence public policy” [32, page 161]. He has tried his model in a secondary school in the USA. His proposed Citizen Action program is a one year-long program in which students would spend most of their study time in this program. The program includes (a) knowledge courses: political-legal process and communications courses; (b) project-based learning: action in literature project and citizen action project, and (c) practicum: community service internship [32, pages 169–177].

 Both Kohlberg and Newmann emphasized on real action. Their proposed approaches are constructive and innovative but very difficult to implement in normal schools. On the other hand, the idea of setting up specific and relevant teaching programs to provide students with first-hand experience of real social action and authentic opportunity to exercise genuine autonomy through behavioral decision making could be an ambitious goal of whole-person education.

## 9. Conclusive Remarks and Suggestions for Future Research

 Behavioral competence is delineated in terms of four parameters: (a) Moral and Social Knowledge, (b) Social Skills, (c) Positive Characters and Positive Attributes, and (d) Behavioral Decision Process and Action Taking. Since Ma's [1–3] previous papers have discussed the moral and social knowledge as well as the social skills in detail, this paper focuses on the last two parameters. We hypothesize that the following twelve positive characters are highly related to behavioral competence: humanity, intelligence, courage, conscience, autonomy, respect, responsibility, naturalness, loyalty, humility, assertiveness, and perseverance. Large-scale future studies should be conducted to substantiate the predictive validity of the complete set of these positive characters. In addition, longitudinal studies are useful to investigate the development of these positive characters and its relationship with social behaviour at different ages. Finally cross-cultural studies should be carried out to test the cultural universality of the twelve positive characters in the prediction of positive or prosocial behaviors in different cultures.

 The whole judgment and behavioral decision process is quite complicated. The direction of future studies should focus more on the complex input, central control, and output subprocesses, and the interactions among these subprocesses. In particular, the impact of an individual's emotional state on the function and implementation of each component of the behavioral decision process is most interesting and important for understanding the real action taking mechanism. The understanding of the formation of behavior is crucial to whole-person education and the study of positive youth development.
